# Desire for reversal after tubal sterilization in the United States, 2006–2023

**DOI:** 10.1016/j.xfre.2026.02.002

**Published:** 2026-03-23

**Authors:** Amy Yunyu Chiang, Aileen M. Gariepy, Daisy Leon-Martinez, Kelly M. Treder, Eleanor Bimla Schwarz

**Affiliations:** a Division of General Internal Medicine, Department of Medicine, University of California San Francisco (UCSF), San Francisco, California; b Department of Obstetrics and Gynecology, Weill Cornell Medicine, Cornell University, New York, New York; c Department of Obstetrics, Gynecology and Reproductive Sciences, School of Medicine, University of California San Francisco (UCSF), San Francisco, California; d Department of Obstetrics, Gynecology, School of Medicine, Boston Medical Center, Boston University, Boston, Massachusetts

**Keywords:** Tubal sterilization, tubal reversal, regret, NSFG, permament contraception, tubal ligation

## Abstract

**Objective:**

To describe trends in the prevalence of tubal sterilization and subsequent desire for reversal among US women and identify sociodemographic factors associated with desires for reversal.

**Design:**

Nationally representative data were collected from US women between 2006 and 2023 in a series of six cross-sectional waves of the National Survey of Family Growth. We employed weighted logistic regression models to assess associations between sociodemographic variables and outcomes of interest.

**Subjects:**

Nationally representative samples of US women aged 15**–**49 surveyed between 2006 and 2023.

**Exposure:**

Sociodemographic variables including age at interview, race and ethnicity, health insurance status, poverty level, education, marital status, parity, and timing of sterilization.

**Main Outcome Measures:**

Tubal sterilization, desire for tubal reversal surgery, and experience with tubal reversal surgery.

**Results:**

In 2022–2023, 21.3% of US individuals who had undergone tubal sterilization reported a desire for reversal. Only 0.1% of those who desired reversal had undergone reversal surgery. Rates of tubal sterilization and desired reversal were higher in prior years; in 2006, a desire for reversal surgery was reported by 26.8% of respondents. Desire for reversal was more likely among nulliparous individuals, those younger than 30 years, those living below the federal poverty level, and among Hispanic and Black women compared with White, non-Hispanic women.

**Conclusion:**

One of every five US individuals who undergo tubal sterilization subsequently reports a desire for reversal. As <1% of those who desire reversal obtain reversal surgery, efforts to support informed decision-making before permanent contraceptive surgery remain important.

Contraceptive tubal surgery, commonly referred to as tubal sterilization, tubal ligation, or permanent contraception, has been the most prevalent form of contraception in the US for many years (1). Since the June 2022 Dobbs decision, requests for tubal sterilization have become more common, with larger increases seen in states with more restrictive abortion policies (2, 3, 4, 5, 6). This growing demand reflects the importance of avoiding undesired pregnancy in an increasingly challenging policy environment.

Although permanent contraception aligns well with some patients’ reproductive goals, reports of regret or desires for reversal after tubal sterilization procedures are troubling and underscore the critical need for thorough presurgical counseling and informed decision-making (7, 8). In some cases, desires for reversal may reflect life changes that shift family preferences. In other situations, desires for reversal may signal inadequate counseling, limited contraceptive options, or decisions made under pressure without voluntary consent. In 1976, in response to rising awareness of involuntary tubal sterilization procedures, a federally mandated consent form for US patients with publicly funded insurance was introduced (9). Form HHS-687 stresses the procedure’s permanence and must be signed by the patient and the counseling clinician, at least 30 days before performing a tubal sterilization. However, form HHS-687 has been criticized for its inappropriately high reading level; in one study, among individuals who had reviewed the federal mandatory consent, 34% still incorrectly believed that contraceptive tubal surgery is reversible (10).

Previous research has highlighted that regret and desires for reversal after tubal sterilization vary by age, with higher levels of regret after procedures performed for younger women (11, 12, 13, 14), and those who had the procedure performed immediately postpartum (15). Contraceptive experiences are also shaped by structural and social factors, including socioeconomic status (16, 17, 18, 19, 20). Some studies have found racial and ethnic variation in rates of regret or desires for reversal after tubal sterilization, with Black and Hispanic women more commonly reporting regret or desires for reversal (16, 17). In this study, we describe temporal changes in rates of tubal sterilization and subsequent desires for reversal among US women using nationally representative data from the National Survey of Family Growth (NSFG), with consideration of variation by socioeconomic status, race, and ethnicity.

## Materials and methods

We conducted a retrospective analysis of publicly accessible data from NSFG. The NSFG is a periodic nationally representative survey conducted to provide estimates of factors affecting pregnancy and birth outcomes in the United States, including sexual activity and contraceptive use (21). The NSFG uses a multistage probability sample designed to represent US women aged 15–44 years, residing in all 50 states and the District of Columbia (22). Interviews were conducted in person by a trained female interviewer in the respondents’ homes. All waves of the NSFG include both a “respondent file,” with information on participants’ demographic characteristics, and a “pregnancy interval file,” with data on each participant’s lifetime pregnancy history and the type of contraception used at the time of each pregnancy. We merged the pregnancy file with the respondent file. The unit of analysis is the female respondent, clustered by the individual to account for multiple pregnancies reported by the same respondent.

We pooled observations from five survey waves: 2006–2010, 2011–2013, 2013–2015, 2015–2017, and 2017–2019. As no NSFG data are available for 2020–2021 due to the COVID epidemic and as the 2022–2023 NSFG restricted use of certain variables (e.g., age at sterilization and timing of sterilization), we analyzed the 2022–2023 NSFG data separately from the prior data. The 2006–2019 file included survey responses from 21,737 female respondents, whereas the 2022–2023 file includes 3,076 female respondents. All analyses used survey weights to account for NSFG’s complex sampling design and oversampling of specific subgroups, ensuring nationally representative estimates. We focused this analysis on respondents who reported a tubal sterilization before their NSFG interview. We identified respondents as having had a tubal sterilization if they answered “yes” to the question “Have you ever had both of your tubes tied, cut, or removed? This procedure is often called a tubal ligation or tubal sterilization.”

### Measures

We defined desires for reversal after tubal sterilization using responses to two items. First, the survey asked if the respondent, “wants to reverse her tubal ligation? (definitely yes, probably yes, probably no, definitely no, or don’t know)” and then a second question asked, “Have you ever had surgery to reverse tubal sterilization? (yes/no).” In keeping with previous work in the field (23), we dichotomized responses by combining “definitely yes” and “probably yes” as indicating desires for reversal, and combining those who said “definitely no” and “probably no” for these analyses. We coded those who stated “don’t know” as having missing data and excluded them from the main analysis to reduce outcome misclassification.

We examined sociodemographic characteristics of survey respondents that may be associated with desires for reversal, including age at interview, poverty level (≤100%, 100%–213%, 213%–400%, and >400% of the federal poverty level), education level (high school or less, some college or more), parity (0 vs. 1–2 births vs. 3+ births), and marital status (married, not married). As NSFG does not include information on health insurance at the time of sterilization, we examined associations with respondents’ health insurance status at the time of interview. The NSFG collects data on health insurance using the following categories: (a) private, (b) Medicaid, Children’s Health Insurance Program or a state-sponsored health plan, (c) Medicare, Military health care or other government health care, and (d) a single service plan, the Indian Health Service or currently not covered by health insurance. We combined NSFG insurance categories (b) and (c) for this analysis. We also considered age at tubal sterilization (categorized as ≤30, >30) (17) and timing of tubal sterilization (postpartum vs. interval). We examined variation by self-reported race and ethnicity using the NSFG’s race and ethnicity categories, which included Hispanic, non-Hispanic White, non-Hispanic Black, and non-Hispanic other or multiple races. The NSFG public use data do not include state of residence or geographical region.

### Statistical analysis

We calculated the weighted proportion of respondents who reported tubal sterilization and regret (i.e., who wanted or had tubal reversal) over the six NSFG survey waves (2006–2010, 2011–2013, 2013–2015, 2015–2017, 2017–2019, and 2022–2023). Survey-weighted cross-tabulations were then conducted to examine the association between sociodemographic variables and time period. Next, we assessed bivariate associations between sterilization regret and sociodemographic factors. Finally, we used multivariate logistic regression models to examine the relationships between sterilization regret, health insurance, and race and ethnicity, adjusting for age at sterilization, age at interview, poverty level, education, parity, marital status, and postpartum sterilization. We included survey wave fixed effects and report adjusted odds ratios (aOR) with 95% confidence intervals (CIs).

Because the primary outcome (sterilization regret) had substantial missingness in 2013–2015 due to a programming error noted by NSFG (24), to identify individual factors associated with regret, we conducted the main analyses using complete-case analysis, restricting the sample to respondents with no missing data (N = 3,344 for 2006–2019, N = 391 for 2022–2023). As a robustness check, we also conducted multiple imputation with chained equations for all variables in the main analytic models (Supplemental Table 1, available online) (25), as well as a sensitivity analysis excluding data from 2013–2015 (Supplemental Table 2). All analyses were performed using STATA 19. The Institutional Review Board of the University of California, San Francisco determined this study to be exempt in review number 23-40339.

## Results

Of all female respondents to the NSFG waves between 2006 and 2023, 21.6% reported having undergone tubal sterilization. The weighted proportion of respondents reporting tubal sterilization declined over time, from 23.7% in 2006–2010 to 16.1% in 2022–2023 (Table 1). Desires for reversal after tubal sterilization also declined, from 26.8% to 21.3% over the same period. Overall, only 0.6% of those who expressed desires for reversal had undergone tubal reversal.

**Table 1:** Tubal sterilization and desire for reversal among female respondents to the National Survey of Family Growth, 2006–2023^a^.

	2006–2010	2011–2013	2013–2015	2015–2017	2017–2019	2022–2023	Total 2006–2023
Female respondents	7,538	3,503	3,476	3,511	3,709	3,076	24,813
Tubal sterilization	23.7%	22.2%	19.5%	23.6%	22.9%	16.1%	21.6%
Desired reversal^b^	26.8%	27.7%	25.0%	26.2%	22.2%	21.3%	24.6%
Definite desire for reversal^c^	13.8%	11.2%	14.5%	16.6%	11.2%	13.5%	13.5%
Missing data^d^ on desires for reversal	17.1%	48.1%	42.0%^e^	17.8%	19.7%	17.8%	26.8%
Tubal reversal completed^e^	1.2%	0.2%	0.5%	0.6%	1.0%	0.1%	0.6%

*Note:* NSFG = National Survey of Family Growth.

a Weighted to reflect the US female population. No NSFG data are available for 2020 or 2021.

b Proportion of those who had a tubal sterilization who answered “definitely yes” or “probably yes” to the question of if the respondent “wants to reverse her tubal ligation”.

c Proportion of those who had a tubal sterilization who answered “definitely yes” when asked if “wants to reverse her tubal ligation?”.

d High missingness in 2013–2015 is due to programming errors noted in the NSFG codebook: https://www.cdc.gov/nchs/data/nsfg/nsfg_2013_2015_userguide_maintext.pdf.

e Proportion of those who desired reversal who obtained tubal reversal surgery.

Between 2006–2019 and 2022–2023, the demographic and socioeconomic profile of women who underwent tubal sterilization remained largely similar, with no statistically significant changes in race/ethnicity, insurance type, age at interview, or parity (Table 2). In the 2022–2023 NSFG, respondents reporting tubal sterilization were more likely to be married than in prior years (76.8% vs. 54.6%, *P*<.001), and to have an annual income of >400% of the federal poverty level (22.5% vs. 11.5%, *P*<.001).

**Table 2:** Characteristics of US women reporting tubal sterilization by time period, 2006–2023^a^.

Characteristics	2006–2019 (N = 3,344)	2022–2023 (N = 391)	*P* value
Race/ethnicity			.44
White, non-Hispanic	52.7	58.9	
Hispanic	23.3	24.6	
Black, non-Hispanic	16.6	9.5	
Other or multiple, non-Hispanic	7.4	7.1	
Health insurance			.50
Private	51.0	53.1	
Medicaid, CHIP or state	23.1	26.2	
Other government, Indian, or no insurance	25.9	20.7	
Age at interview			.62
≤30	12.0	10.1	
>30	88.0	89.9	
Poverty level			<.001
≤100%	33.5	20.4	
100%–213%	28.5	33.7	
213%–400%	26.5	23.4	
>400%	11.5	22.5	
Parity			.22
None	0.87	0.26	
1 or 2	41.4	42.8	
3 or more livebirths	57.8	56.9	
Married			<.001
No	45.4	23.2	
Yes	54.6	76.8	
Age at sterilization^b^			
≤30	32.7	n/a	
>30	67.3	n/a	
Timing of sterilization^b^			
Interval	67.5	n/a	
Postpartum	32.5	n/a	

*Note:* CHIP = Children’s Health Insurance Program; NSFG = National Survey of Family Growth.

a Percentages weighted to reflect the US female population.

b Publicly available 2022–2023 NSFG data do not include variables on age at sterilization or timing of sterilization.

From 2006 to 2019, the desire for reversal was more likely among Hispanic and Black women compared with White, non-Hispanic women, even after adjusting for sociodemographic factors (Table 3). Women under age 30, those with lower income, less education, and those who were unmarried or nulliparous also had higher odds of desires for reversal. Respondents with the highest household incomes had significantly lower odds of desires for reversal than those living below the federal poverty level, as did those with higher parity.

**Table 3:** Sociodemographic variables associated with desire to reverse tubal sterilization, 2006–2023^a^.

Characteristics	2006–2019		2022–2023	
	Unadjusted (N = 3,344)	Adjusted^b^ (N = 3,344)	Unadjusted (N = 391)	Adjusted^b^ (N = 387)
Parity				
Nulliparous	Reference	Reference	Reference	Reference
1 or 2	0.11∗∗	0.06∗∗	0.70	0.83
	(0.06–0.22)	(0.03–0.15)	(0.33–1.45)	(0.42–1.66)
3 or more livebirths	0.15∗∗	0.07∗∗	–	–
	(0.08–0.29)	(0.03–0.19)	–	–
Age at interview				
≤30	2.07∗∗	1.61	1.38	0.88
	(1.40–3.06)	(1.01–2.55)	(0.52–3.63)	(0.27–2.85)
>30	Reference	Reference	Reference	Reference
Race and ethnicity				
White, non-Hispanic	Reference	Reference	Reference	Reference
Hispanic	1.57∗∗	1.38∗	2.49∗	2.27∗∗
	(1.21–2.03)	(1.00–1.89)	(1.18–5.26)	(1.00–5.17)
Black, non-Hispanic	1.97∗∗	1.73∗∗	3.14∗	2.57
	(1.34–2.91)	(1.18–2.54)	(1.26–7.81)	(0.85–7.72)
Other or multiple, non-Hispanic	1.41	1.39	2.48∗∗	3.38∗
	(0.65–3.08)	(0.635–2.95)	(0.98–6.29)	(1.23–9.26)
Health insurance				
Private	Reference	Reference	Reference	Reference
Medicaid, CHIP and other state-sponsored	1.45∗∗	1.06	2.53∗	1.48
	(0.95–2.23)	(0.69–1.66)	(1.14–5.60)	(0.52–4.22)
Other government plan or no insurance	1.74∗∗	1.32	1.49	0.78
	(1.17–2.60)	(0.85–2.05)	(0.60–3.69)	(0.27–2.21)
Poverty level				
≤100%	Reference	Reference	Reference	Reference
100%–213%	0.96	1.15	0.87	1.13
	(0.72–1.29)	(0.87–1.53)	(0.39–1.94)	(0.44–2.85)
213%–400%	0.72	1.09	0.35	0.55
	(0.44–1.17)	(0.67–1.79)	(0.10–1.19)	(0.14–2.13)
>400%	0.33∗∗	0.43∗	0.22∗∗	0.45
	(0.20–0.53)	(0.21–0.87)	(0.09–0.58)	(0.11–1.76)
Education				
High school or less	Reference	Reference	Reference	Reference
Some college or more	0.67∗∗	0.89	0.62	0.87
	(0.52–0.86)	(0.67–1.20)	(0.35–1.11)	(0.46–1.66)
Married				
No	Reference	Reference	Reference	Reference
Yes	0.67∗	0.86	0.43∗	0.64
	(0.49–0.91)	(0.64–1.15)	(0.22–0.83)	(0.30–1.36)
Age at sterilization^c^				
≤30	2.62∗∗	2.12∗∗	–	–
	(1.89–3.65)	(1.47–3.07)		
>30	Reference	Reference	–	–
Timing of sterilization^c^				
Interval	Reference	Reference	–	–
Postpartum	1.15	1.11	–	–
	(0.82–1.62)	(0.77–1.59)	–	–

*Note:* CHIP = Children’s Health Insurance Program; NSFG = National Survey of Family Growth.

a Weighted to reflect the US female population; no NSFG data are available for 2020–2021.

b Adjusted for all variables shown in the table.

c Age at sterilization and timing of sterilization were not able to be generated in the NSFG 2022–23 file due to restricted use. We therefore pooled observations from the first five survey waves (2006–2010, 2011–2013, 2013–2015, 2015–2017, 2017–2019) and analyzed the 2022–2023 NSFG data separately from the prior data.

∗∗ *P*<.01.

∗ *P*<.05.

In 2022–2023, these associations generally held, although CIs widened due to a smaller sample size. Hispanic and Black women continued to exhibit higher odds of desires for reversal, with point estimates even larger than in earlier years (Table 3). Non-Hispanic women of other or multiple races had significantly higher odds of desires for reversal compared with White women (aOR = 3.38, 95% CI: 1.23–9.26). Medicaid recipients more often reported desires for reversal in unadjusted models, but this association was attenuated and not significant in the multivariate model.

Results from the imputed models (Supplemental Table 1) between the two periods were consistent with the main analyses. Results from the sensitivity analysis excluding data from the 2013–2015 wave (which had higher-than-usual rates of missing data) were also generally consistent (Supplemental Table 2), but some associations were slightly attenuated. Non-Hispanic Black respondents continued to show significantly higher odds of desires for reversal compared with non-Hispanic White (aOR = 1.70, 95% CI: 1.13–2.57), whereas Hispanic respondents were not significantly more likely to report desires for reversal in adjusted models.

## Discussion

This analysis of nationally representative surveys of US women found that rates of both tubal sterilization and desires for reversal have decreased in recent years. However, in the most recent NSFG data, desire for reversal was reported by almost one in every five of those who underwent tubal sterilization. As sterilization regret has been linked to depressive symptoms and adverse mental health outcomes (26), it can meaningfully detract from a patient’s quality of life. However, desires for reversal must be understood not just as an individual outcome but within the context of structural inequities that impact reproductive autonomy. Concerns of reproductive coercion, including forced sterilizations of Black, Latina, and Indigenous women, have eroded trust in the medical establishment and continue to shape both contraceptive decisions and regrets (27, 28). Systemic barriers to person-centered contraceptive care include lack of universal access to health care in the US, and Medicaid’s 30-day waiting period, which makes the timing of tubal sterilization a constrained choice for low income patients rather than a fully autonomous decision (9, 29).

Limited information about alternative contraceptive options has been associated with a higher prevalence of regrets. (13, 30, 31, 32) Regrets appear to be less common after a vasectomy which is easier to reverse (33, 34). Prior studies have shown that many women who experience regret after tubal sterilization lacked adequate knowledge about the procedure’s permanence or were unaware of alternative contraceptive options (7, 15). In one study, 28.6% of patients who had previously undergone tubal sterilization reported not discussing alternatives to this surgery with their clinician (15). This underscores the need for high-quality, person-centered care that respects each individual’s values, future goals, and lived context. Cultural expectations around motherhood, family pressures, and economic instability may amplify regret, particularly among younger women whose desires about childbearing may change as they age (35). Additionally, social support systems (7) and systemic inequities in healthcare access and quality of contraceptive counseling may further shape healthcare and contraceptive experiences.

Although the 2022–2023 NSFG data indicate that fewer women underwent tubal sterilization than in prior years, which may reflect the ways the COVID-19 pandemic limited access to operating rooms, since the Dobbs decision in 2022, there have been multiple reports of increasing rates of tubal sterilization (2, 3, 4, 5, 6). In one study, the increase in permanent contraceptive procedures for women was double that for men (4). Therefore, ongoing monitoring is needed, expecting that as tubal sterilization becomes more common, efforts to safeguard against regret may become more urgent.

A key strength of this study is the use of a large, nationally representative sample, which allows for robust analyses of sociodemographic variation in sterilization regret and comparison of trends over time. However, several limitations should be noted. The NSFG data do not distinguish between bilateral salpingectomy and other approaches to tubal sterilization. It is possible that lower rates of desire for reversal may reflect understanding that reversal is not possible after bilateral salpingectomy, growing appreciation of salpingectomy’s ability to reduce ovarian cancer originating in the distal portion of the fallopian tube, or the increasing success of in vitro fertilization without tubal reversal.

Missing data on desire for reversal after some tubal sterilizations limit the precision of population estimates. The cross-sectional nature of the study limits causal interpretations, underscoring the need for longitudinal research to track women’s experiences before and after sterilization over time, understanding that feelings of regret and desires for reversal may change over time. The reliance on self-reported data introduces the potential for recall and social desirability biases, which may influence responses. Additionally, although the study adjusts for multiple sociodemographic variables, unmeasured factors—such as access to reproductive healthcare, quality of contraceptive counseling, provider biases, and cultural influences remain. We recognize that the smaller sample size in the 2022–2023 data limited power when considering some associations. Finally, findings may not be generalizable to populations with universal access to healthcare.

## Conclusion

In conclusion, one of every five patients in the US who undergo tubal sterilization later report a desire for tubal reversal surgery, which very few patients are able to obtain, requiring consideration of in vitro fertilization. Although it is heartening to note some reductions in desires for reversal after tubal sterilization in recent years, efforts are needed to ensure patients considering these procedures clearly understand that it is a permanent method of contraception. Careful consideration of the complex interplay of social factors in shaping contraceptive decision-making is warranted alongside efforts to eliminate structural barriers to comprehensive healthcare.

## CRediT Authorship Contribution Statement

**Amy Yunyu Chiang:** Writing – original draft, Methodology, Formal analysis, Conceptualization. **Aileen M. Gariepy:** Writing – review & editing. **Daisy Leon-Martinez:** Writing – review & editing. **Kelly M. Treder:** Writing – review & editing. **Eleanor Bimla Schwarz:** Writing – review & editing, Supervision, Funding acquisition, Conceptualization.

## Declaration of Interests

A.Y.C. reports funding from Agency for Health Care Research and Quality, R18 for the submitted work. A.M.G. has nothing to disclose. D.L.-M. reports funding from Winn Career Development Award (10/2023—1/2026) outside the submitted work. K.M.T. reports funding from Patient-Centered Outcomes Research Institute, Disseminating the safety and effectiveness of tubal ligation compared to intrauterine contraception Contract #DI-2023C3-35486 outside the submitted work. E.B.S. reports funding from Agency for Health Care Research and Quality, R18 for the submitted work; funding from Patient-Centered Outcomes Research Institute, Disseminating the safety and effectiveness of tubal ligation compared to intrauterine contraception Contract #DI-2023C3-35486 outside the submitted work.

## References

[1] Daniels K., Abma J.C. Current contraceptive status among women aged 15–49: United States, 2017–2019. https://www.cdc.gov/nchs/data/databriefs/db388-H.pdf.

[2] Xu X., Chen L., Desai V.B., Gross C.P., Pollack C.E., Schwartz P.E. (2024). Tubal sterilization rates by state abortion laws after the Dobbs decision. J Am Med Assoc.

[3] Strasser J., Schenk E., Luckenbill S., Tsevat D., King L., Luo Q. (2025). Tubal sterilization and vasectomy increased among US young adults after the Dobbs Supreme Court decision In 2022. Health Aff (Millwood).

[4] Ellison J.E., Brown-Podgorski B.L., Morgan J.R. (2024). Changes in permanent contraception procedures among young adults following the Dobbs decision. J Am Med Assoc Health Forum.

[5] Mitchell J.A., Yao M., Maeda R., Lappen J.R., Brant A.R. (2024). Permanent and long-acting reversible contraception volumes at a multihospital system in Ohio before and after Dobbs. Contraception.

[6] Lemieux M., Zhou C., Cary C., Kelly J. (2024). Changes in reproductive health information-seeking behaviors after the Dobbs decision: systematic search of the Wikimedia database. JMIR infodemiology.

[7] Siemons S.E., Vleugels M.P.H., van Balken M.R., Braat D.D.M., Nieboer T.E. (2022). Male or female sterilization - the decision making process: counselling and regret. Sex Reprod Healthc.

[8] Mosley E.A., Monaco A., Zite N., Rosenfeld E., Schablik J., Rangnekar N. (2023). U.S. physicians’ perspectives on the complexities and challenges of permanent contraception provision. Contraception.

[9] Amalraj J., Arora K.S. (2022). Ethics of a mandatory waiting period for female sterilization. Hastings Cent Rep.

[10] Zite N.B., Philipson S.J., Wallace L.S. (2007). Consent to sterilization section of the Medicaid-Title XIX form: is it understandable?. Contraception.

[11] Curtis K.M., Mohllajee A.P., Peterson H.B. (2006). Regret following female sterilization at a young age: a systematic review. Contraception.

[12] Hillis S.D., Marchbanks P.A., Tylor L.R., Peterson H.B. (1999). Poststerilization regret: findings from the United States collaborative review of sterilization. Obstet Gynecol.

[13] Boring C.C., Rochat R.W., Becerra J. (1988). Sterilization regret among Puerto Rican women. Fertil Steril.

[14] Danvers A.A., Evans T.A. (2022). Risk of sterilization regret and age: an analysis of the National Survey of Family Growth, 2015–2019. Obstet Gynecol.

[15] Rodowa M.S., Waddington A., Pudwell J. (2024). Regret in the modern contraceptive landscape: evaluating regret in patients undergoing tubal ligation or bilateral salpingectomy for contraception. J Obstet Gynaecol Can.

[16] Borrero S., Abebe K., Dehlendorf C., Schwarz E.B., Creinin M.D., Nikolajski C. (2011). Racial variation in tubal sterilization rates: role of patient-level factors. Fertil Steril.

[17] Garcia-Alexander G., Gonzales K.L., Ferguson L.E., Hauck E. (2019). Racial and ethnic disparities in desire for reversal of sterilization among U.S. women. J Womens Health (Larchmt).

[18] Platz-Christensen J.J., Tronstad S.E., Johansson O., Carlsson S.A. (1992). Evaluation of regret after tubal sterilization. Int J Gynaecol Obstet.

[19] Grady C.D., Schwarz E.B., Emeremni C.A., Yabes J., Akers A., Zite N. (2013). Does a history of unintended pregnancy lessen the likelihood of desire for sterilization reversal?. J Womens Health (Larchmt).

[20] Clark S., Levy Z. (2025). Sterilization, infecundity, and reproductive autonomy in rural, suburban, and urban America: results from a national survey. Perspect Sex Reprod Health.

[21] Groves R.M., Benson G., Mosher W.D., Rosenbaum J., Granda P., Axinn W. (2005). Plan and operation of cycle 6 of the National Survey of Family Growth. Vital Health Stat.

[22] Lepkowski J.M., Mosher W.D., Davis K.E., Groves R.M., van Hoewyk J., Willem J. (2006). National Survey of Family Growth, cycle 6: sample design, weighting, imputation, and variance estimation. Vital Health Stat.

[23] Borrero S.B., Reeves M.F., Schwarz E.B., Bost J.E., Creinin M.D., Ibrahim S.A. (2008). Race, insurance status, and desire for tubal sterilization reversal. Fertil Steril.

[24] US Department of Health and Human Services, Public Use Data File Documentation (2017). 2013–2015 National Survey of Family Growth, User’s Guide. June 2025;21. https://www.cdc.gov/nchs/nsfg/nsfg_2013_2015_questionnaires.htm.

[25] Allison P.D. (2002). Quantitative Applications in the Social Sciences.

[26] Shreffler K.M., Greil A.L., McQuillan J., Gallus K.L. (2016). Reasons for tubal sterilisation, regret and depressive symptoms. J Reprod Infant Psychol.

[27] Roberts D. (1997). Killing the black body: race, reproduction, and the meaning of liberty.

[28] Stern A.M. (2016).

[29] Maykin M.M., Pilliod R.A., Werner E.F. (2021). Discriminatory regulations on postpartum sterilization for Medicaid recipients propagate health inequities. Lancet Reg Health Am.

[30] American College of Obstetricians and Gynecologists’ Committee on Practice Bulletins—Gynecology (2019). ACOG Practice Bulletin No. 208: benefits and risks of sterilization. Obstet Gynecol.

[31] Hardy E., Bahamondes L., Osis M.J., Costa R.G., Faúndes A. (1996). Risk factors for tubal sterilization regret, detectable before surgery. Contraception.

[32] Neuhaus W., Bolte A. (1995). Prognostic factors for preoperative consultation of women desiring sterilization: findings of a retrospective analysis. J Psychosom Obstet Gynaecol.

[33] Charles D.K., Anderson D.J., Newton S.A., Dietrich P.N., Sandlow J.I. (2023). Vasectomy regret among childless men. Urology.

[34] Jamieson D.J., Kaufman S.C., Costello C., Hillis S.D., Marchbanks P.A., Peterson H.B. (2002). A comparison of women’s regret after vasectomy versus tubal sterilization. Obstet Gynecol.

[35] Collins P.H. (1995). Justice and care.

