# Supplementary material for: Desire for reversal after tubal sterilization in the United States, 2006–2023
Source: F S Rep. 2026 Mar 23;7(2):143–9. doi: 10.1016/j.xfre.2026.02.002 (PMC13107036; doi:10.1016/j.xfre.2026.02.002)
Supplement: Supplemental Table [file mmc1.docx]

**Appendix**

**Supplemental Table S1 Robustness Check using multiple imputation: Unadjusted and Adjusted Odds of Desire for Reversal of Sterilization ^a,b^**

|  | 2006-2019 | | 2022-2023 | |
| --- | --- | --- | --- | --- |
|  | Unadjusted model with multiple imputation | Adjusted model with multiple imputation | Unadjusted model with multiple imputation | Adjusted model with multiple imputation |
|  | N=4,569 | N=4,568 | N=473 | N=467 |
| **Race/ethnicity** |  |  |  |  |
| White, non-Hispanic | Reference | Reference | Reference | Reference |
|  |  |  |  |  |
| Hispanic | 1.50** | 1.35+ | 2.41* | 2.18+ |
|  | (1.14 - 1.99) | (1.00 - 1.84) | (1.11 - 5.24) | (0.97 - 4.92) |
| Black, non-Hispanic | 1.87** | 1.64** | 2.70* | 2.33 |
|  | (1.33 - 2.62) | (1.14 - 2.36) | (1.08 - 6.79) | (0.82 - 6.60) |
| Other or multiple, non-Hispanic | 1.33 | 1.31 | 2.53 | 3.20+ |
|  | (0.69 - 2.58) | (0.68 - 2.52) | (0.81 - 7.83) | (1.00 - 10.36) |
| **Health insurance** |  |  |  |  |
| Private | Reference | Reference | Reference | Reference |
|  |  |  |  |  |
| Medicaid, CHIP and other state-sponsored | 1.58** | 1.15 | 2.56* | 1.40 |
|  | (1.13 - 2.20) | (0.77 - 1.71) | (1.17 - 5.59) | (0.51 - 3.89) |
| Other government plan or no insurance | 1.73** | 1.28 | 1.58 | 0.81 |
|  | (1.27 - 2.37) | (0.90 - 1.84) | (0.65 - 3.88) | (0.30 - 2.19) |
| **Age at interview** |  |  |  |  |
| <=30 | 2.06** | 1.56* | 1.73 | 1.17 |
|  | (1.48 - 2.86) | (1.05 - 2.30) | (0.49 - 6.09) | (0.28 - 4.84) |
| >30 | Reference | Reference | Reference | Reference |
| **Poverty level** |  |  |  |  |
| <=100% | Reference | Reference | Reference | Reference |
|  |  |  |  |  |
| 100-213% | 0.91 | 1.12 | 0.85 | 1.07 |
|  | (0.68 - 1.22) | (0.81 - 1.54) | (0.37 - 1.95) | (0.40 - 2.86) |
| 213-400% | 0.66+ | 1.00 | 0.35 | 0.55 |
|  | (0.45 - 0.99) | (0.64 - 1.58) | (0.11 - 1.10) | (0.15 - 1.99) |
| >400% | 0.31** | 0.48* | 0.25** | 0.50 |
|  | (0.19 - 0.51) | (0.25 - 0.90) | (0.08 - 0.73) | (0.12 - 2.18) |
| **Education** |  |  |  |  |
| High school or less | Reference | Reference | Reference | Reference |
|  |  |  |  |  |
| Some college or more | 0.67** | 0.90 | 0.6 | 0.82 |
|  | (0.51 - 0.88) | (0.67 - 1.21) | (0.33 - 1.12) | (0.41 - 1.65) |
| **Parity** |  |  |  |  |
| None | Reference | Reference | Reference | Reference |
|  |  |  |  |  |
| 1 or 2 | 0.21** | 0.12** | 0.76 | 0.88 |
|  | (0.09 - 0.48) | (0.05 - 0.27) | (0.37 - 1.57) | (0.43 - 1.79) |
| 3 or more livebirths | 0.28** | 0.14** | - | - |
|  | (0.13 - 0.63) | (0.06 - 0.33) | - | - |
| **Married** |  |  |  |  |
| No | Reference | Reference | Reference | Reference |
|  |  |  |  |  |
| Yes | 0.66** | 0.87 | 0.45* | 0.68 |
|  | (0.51 - 0.85) | (0.66 - 1.14) | (0.24 - 0.86) | (0.31 - 1.50) |
| **Age at sterilization** |  |  |  |  |
| <=30 | 1.91** | 1.73** | - | - |
|  | (1.45 - 2.50) | (1.27 - 2.35) |  |  |
| >30 | Reference | Reference | - | - |
| **Timing of sterilization** |  |  |  |  |
| Interval | Reference | Reference | - | - |
|  |  |  |  |  |
| Postpartum | 1.22+ | 0.89 | - | - |
|  | (0.97 - 1.55) | (0.68 - 1.16) | - | - |

^a Adjusted for all other covariates in the table.^

^b Age at sterilization and timing of sterilization were not able to be generated in the NSFG 2022-23 file due to restricted use. We therefore pooled observations from first five survey waves (2006-2010, 2011-2013, 2013-2015, 2015-2017, 2017-2019) and analyzed the 2022-2023 NSFG data separately from the prior data. no data was collected by NSFG between 2020-2021.^

^** p<0.01 *p<0.05 + p<0.1^

**Supplemental Table S2 Sensitivity Analysis: Excluding 2013-2015, when calculating Unadjusted and Adjusted Odds of Desire for Sterilization Reversal ^a^**

|  | 2006-2019, excluding 2013-2015 | |
| --- | --- | --- |
|  | Unadjusted | Adjusted |
|  | N=2,996 | N=2,996 |
| **Race/ethnicity** |  |  |
| White, non-Hispanic | Reference | Reference |
|  |  |  |
| Hispanic | 1.51** | 1.30 |
|  | (1.13 - 2.02) | (0.90 - 1.88) |
| Black, non-Hispanic | 1.95** | 1.70* |
|  | (1.28 - 2.97) | (1.13 - 2.57) |
| Other or multiple, non-Hispanic | 1.49 | 1.38 |
|  | (0.62 - 3.61) | (0.59 - 3.24) |
| **Health insurance** |  |  |
| Private | Reference | Reference |
|  |  |  |
| Medicaid, CHIP and other state-sponsored | 1.40 | 1.02 |
|  | (0.86 - 2.27) | (0.63 - 1.67) |
| Other government plan or no insurance | 1.63* | 1.27 |
|  | (1.04 - 2.55) | (0.78 - 2.07) |
| **Age at sterilization** |  |  |
| <=30 | 2.29** | 1.82** |
|  | (1.60 - 3.29) | (1.25 - 2.65) |
| >30 | Reference | Reference |
|  |  |  |
| **Age at interview** |  |  |
| <=30 | 2.17** | 1.80* |
|  | (1.40 - 3.36) | (1.09 - 2.97) |
| >30 | Reference | Reference |
|  |  |  |
| **Timing of sterilization** |  |  |
| Interval | Reference | Reference |
|  |  |  |
| Postpartum | 1.16 | 1.09 |
|  | (0.78 - 1.72) | (0.72 - 1.64) |
| **Poverty level** |  |  |
| <=100% | Reference | Reference |
|  |  |  |
| 100-213% | 0.98 | 1.15 |
|  | (0.71 - 1.34) | (0.86 - 1.54) |
| 213-400% | 0.74 | 1.09 |
|  | (0.43 - 1.26) | (0.64 - 1.86) |
| >400% | 0.33** | 0.38* |
|  | (0.18 - 0.56) | (0.16 - 0.86) |
| **Education** |  |  |
| High school or less | Reference | Reference |
|  |  |  |
| Some college or more | 0.71** | 0.90 |
|  | (0.54 - 0.93) | (0.65 - 1.24) |
| **Parity** |  |  |
| None | Reference | Reference |
|  |  |  |
| 1 or 2 | 0.10** | 0.05** |
|  | (0.05 - 0.21) | (0.02 - 0.14) |
| 3 or more livebirths | 0.15** | 0.07** |
|  | (0.08 - 0.29) | (0.02 - 0.19) |
| **Married** |  |  |
| No | Reference | Reference |
|  |  |  |
| Yes | 0.70* | 0.88 |
|  | (0.50 - 0.98) | (0.64 - 1.21) |

^a Adjusted for all other covariates in the table.^

^** p<0.01 *p<0.05 + p<0.1^
